# Fatigue dataset for carbon fibre-reinforced polymers under uni- and multiaxial loads with varying biaxiality and proportional stress ratios

**DOI:** 10.1016/j.dib.2022.108757

**Published:** 2022-11-18

**Authors:** Marc Moeller, Jochen Blaurock, Gerhard Ziegmann

**Affiliations:** a University of Applied Sciences Cologne, Institute for Automotive Engineering, Betzdorfer Str. 2, 50679 Köln; b Clausthal University of Technology, Institute of Polymer Materials and Plastics Engineering, Agricolastraße 6, 38678 Clausthal-Zellerfeld

**Keywords:** Multiaxial fatigue, Composites, Carbon-fibre, Reinforced materials, Lifetime, Proportional loads

## Abstract

The experimental data in this submission is related to parts of the dissertation “Bruchkurvenmodellierung von kohlenstofffaserverstärkten Kunststoffen bei mehrachsig nichtproportionaler Ermüdungsbeanspruchung” [1] and partly to further publications on the fatigue of carbon fibre-reinforced plastic [2]. The background to the experimental investigations is the need to better understand the behavior of fiber-reinforced plastics under various multiaxial loads. For this purpose, comprehensive tests ranging from uniaxial tests on unidirectional up to multiaxial tests on multidirectional specimens are conducted. The details in this paper include the preparation of specimens, the chemical compositions, the stresses based on each single specimen's geometry and the test settings of the cyclic experiments. In this article, the experimental data for failure and number of endured cycles under uni- and proportional multiaxial loads are explained in detail, while additionally, all the raw experimental data on the hysteresis loops can be found in the Mendeley Data repository [3]. The dataset can therefore be used for their own validations and analysis, and it allows the reader to trace back all the settings made during the tests. The manufacturing of the carbon fibre-reinforced tube specimen by filament winding is carried out at the Institute of Polymer Materials and Plastics Engineering of the Clausthal University of Technology and the cyclic tension- or compression-torsion tests are conducted on a servo-hydraulic testing machine at the Institute of Automotive Engineering (IFK) of TH Köln – University of Applied Sciences. Radially wound quasi-unidirectional tube test specimens with the layer structure [±90], balanced angle-ply laminates with the layer structure [±70] and multidirectional laminates with the layer structure [90/±70] were used. The investigated material system consists of carbon fiber-reinforced plastic with a thermoset epoxy resin as the matrix material.

**Specifications Table**

**Table:** 

Subject	Polymers and Plastics
Specific subject area	Fatigue, Composites, Multiaxial Loads.
Type of data	Table
How the data were acquired	• The cyclic tension/compression-torsion tests are performed on a servo-hydraulic Instron\® 8802 testing machine with ○ a hydrostatic axial cylinder with 100 kN nominal force at 210 bar and a nominal stroke of ±75 mm and ○ a torsion cylinder with 1.000 Nm nominal torque at 210 bar, a nominal angle of rotation of ±45°. • System and Transducer measurement accuracies: ○ The displacement is measured by an integrated displacement transducer LVDT (Linear Variable Differential Transformer) and the rotatory angle is measured by an integrated angle of rotation transducer with ±0.2 % of Transducer full travel. ○ Force and torque are measured by a biaxial load cell with an axial force of 160 kN and a torsional moment of 1000 Nm with an accuracy class of the combined force-torsion transducer of ±0.002 % of the load cell capacity or ±0.5 % of the indicated load, whichever is greater (Meets or Surpasses IS07500-1 Class 0.5, ASTM E 4, EN10002-2 Class 0.5). ○ Data Acquisition rate of the system used is 10 kHz. • The tube specimen are filament winded with carbon fibre rovings Tenax®E HTS45 of the standard module class and with the medium-viscosity, unmodified epoxy resin Araldite® LY 556 by Huntsman, which is based on bisphenol-A. For a secure clamping the specimen are equipped with an aluminum insert in the clamping area and then hydraulically clamped with interchangeable collets for tubular specimen with outer diameters up to 64 mm.
Data format	Raw
Description of data collection	The uniaxial tests were performed with pulsating (R=0.1, R=0.5) or fully-reversed (R=-1) stress ratios for either force- or torque-controlled cyclic loading with constant amplitude of carbon fibre-reinforced plastic (CFRP) tube specimen. In the case of the multiaxial tests, only results for proportional loading, which are simultaneously force- and torque-controlled with the same frequency and stress ratio, are referred to in this article. In terms of operational stability, the tests have been carried out on several different force- and/or torque-levels and not always but partly multiple times.
Data source location	• University of Applied Sciences Cologne • Faculty of Automotive Systems and Production • Institute for Automotive Engineering • Cologne • Germany • Betzdorfer Str. 2 • 50679 Köln
Data accessibility	Partial data (with information on the specimen, test conditions and endured number of cycles) is presented in a summary document and also the detailed raw data with information per cycle (hysteresis loops) is in mendeley data repository [3]: DOI: 10.17632/jpk2t755vg Direct URL to data: https://doi.org/10.17632/jpk2t755vg.1
**Related research article**	

## Value of the Data

• The acquired data can be used to validate or (further) develop failure criteria for fatigue of fibre-reinforced plastics. It can also be useful for evaluating the extent of simultaneous occurrence of multiaxial fatigue in composites.
• Scientists or developers on national, regional and global scales, who want to validate their mathematical models on experimental data or create models based on existing experimental data can benefit from the data.
• Based on the experiments performed, further experiments can be developed, using the existing data to e.g. base further combinations of stresses on various stress ratios and/or on other combinations of tension/compression-torsional stresses.

## 1 Data Description

The uniaxial and multiaxial tests were performed with pulsating (R=0.1, R=0.5) or fully-reversed (R=-1) stress ratios for either or both force- and/or torque-controlled cyclic loading with constant amplitude. In the case of the axial force, the stress ratio is referred to as $R_{A}$ and in the case of torque, the stress ratio is referred to as $R_{T}$. In the case of the multiaxial tests, only results for proportional loading, which are simultaneously force- or torque-controlled with the same stress ratio, are refered to in this article (Part 1). For the multiaxial tests, the biaxiality angle between torsional and axial load is considered as

$$\xi =tan^{-1}\left(\frac{\tau_{max}}{\sigma_{max}}\right)$$

Therefore, the normal and tangential stresses are calculated in terms of the axial normal stress

$$\sigma_{max}=\frac{F_{max}}{A}$$

with $F_{max}$ being the maximum force within a cycle and A the cross-sectional area of each specimen, as well as the shear stress with the use of bredt's formula for thin-walled structures

$$\tau_{max}=\frac{M_{t,max}}{2\cdot A_{m}\cdot t}$$

with the maximum torsional moment within a cycle $M_{t}$, the enclosed area $A_{m}$ and the thickness t of each specimen. In this article, the calculations are used only for the explanations of the specimen naming and the calculation of the biaxiality angle and therefore added to the data in the summary tables in the repository. No other calculations or interpretations are made with the use of these stress calculations in this article. The original forces and moments can be found in the raw data in the mendeley repository [3], for own calculations or interpretations. In terms of operational stability, the tests have been carried out on several different force- or torque-levels, not always but partly multiple times for each level. For each test, the cyclic force-displacement and torque-degree responses in terms of force and torque as well as relative axial and rotatory position of the specimen was conducted within 10 measuring points over a quarter sine wave (40 data points within one cycle), for:

• all cycles in $log_{10}$ increments (Name.steps.tracking.csv in [3])
• the last 10 cycles before failure in detail (Name.Stop.csv in [3]).

**Fig. 1:**
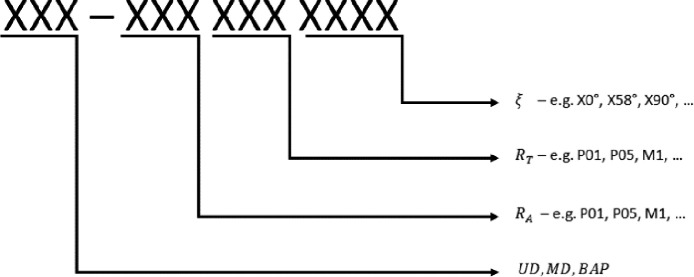
Details on the declaration of the test specimen groups and assignment of the test specimen architecture to the biaxiality and stress ratios of the respective tests.

All of the mentioned raw data has been made available in the Mendeley Data repository [3]. For all data, the declaration and naming of the test groups in the tables as well as the raw data files in the repository is according to the structure shown in Fig. 1.

Wherein the first part is naming the fibre architecture (UD: Unidirectional, MD: Multidirectional, BAP: Balanced Angle-Ply). The second part refers to the stress ratio of the axial force, the third part refers to the stress ratio (P01: R=0.1, P05: R=0.5, M1: R=-1.0) of the torque and the last part refers to the biaxiality angle between torsional and axial load in each group. For uniaxial loads, only one stress ratio is required, which means that the other will be replaced by "XXX" always.

For all the CFRP specimens, the fibre volume fraction of fibre to resin is calculated by the weight of each specimen and the known amount of fibers added by the filament winding of the specimen and added to the table as additional information. In the summary document in the repository [3], the chemical composition of the resin used in the filament winding process is also added by the weight of resin Araldite® LY556, Hardener Aradur 917 and Accelerator DY070 before the merging of the three components. The Cure and Post-Cure times of each shaft is given in the table as well.

The chemical composition is given in tabular form for each shaft in table 12 of the summary document [3], from which several specimens were taken. Within the tables for the fatigue data (Table 1 to Table 11 in the summary document [3]) the origin of each sample is given as well. Within the tables there are multiple abbreviations to clearly arrange the tables:

• No. Number of the Specimen
• SNo. Shaft number of filament winded shaft (origin of specimen)
• φ Fibre-Volume-Fraction for the specimen
• $R_{A}$ Axial stress ratio
• $R_{T}$ Torsional stress ratio
• ξ Biaxiality angle between shear and axial stress
• $f_{A}$ Frequency of the applied axial load
• $f_{T}$ Frequency of the applied torsional load
• N Endured number of cycles until final global failure of the specimen

**Table 1:** Summary of data uploaded to the Mendeley data repository [3] with classification according to Fig. 1.

Configuration	Lay-up	$R_{A}$	$R_{T}$	ξ	φ	No. of Specimen	Table in Summary [3]
	∘	-	-	∘	-	-	
Unidirectional (DU) / uniaxial							
UD-XXXP01×90	${\left[90\right]}_{s}$	-	0.1	90	0,55	27	1
UD-XXXP05×90	${\left[90\right]}_{s}$	-	0.5	90	0,51	9	2
UD-XXXM1×90	${\left[90\right]}_{s}$	-	-1	90	0,53	8	3
UD-P01XXXX0	${\left[90\right]}_{s}$	0.1	-	0	0,51	20	4
UD-M1XXXX0	${\left[90\right]}_{s}$	-1	-	0	0,52	8	5
Unidirectional (UD) / multiaxial / proportional							
UD-P01P01×28	${\left[90\right]}_{s}$	0.1	0.1	28	0,52	13	6
UD-P01P01×58	${\left[90\right]}_{s}$	0.1	0.1	58	0,51	14	7
Balanced Angle-Ply (BAP) / multiaxial / proportional							
BAP-P01P01×19	${\left[\pm 70\right]}_{s}$	0.1	0.1	19	0,55	8	8
BAP-P01P01×49	${\left[\pm 70\right]}_{s}$	0.1	0.1	49	0,55	6	9
Multidirectional (MD) / multiaxial / proportional							
MD-P01P01×28	${\left[90/\pm 70\right]}_{s}$	0.1	0.1	28	0,52	11	10
MD-P01P01×67	${\left[90/\pm 70\right]}_{s}$	0.1	0.1	67	0,53	6	11

In the case of a Prefix before the number of endured cycles, there is some additional information:

• XC Specimen pre-failed inside the clamping or the tapering area
• RO Specimen is stopped and counted as a runout
• HA Specimen was restarted at a higher amplitude after runout

While only the main information on the number of endured cycles for each specimen of each category is shown in the tables, the very detailed hysteresis information can also be found in the mendeley data repository [3].

## 2 Experimental Design, Materials and Methods

**Fig. 2:**
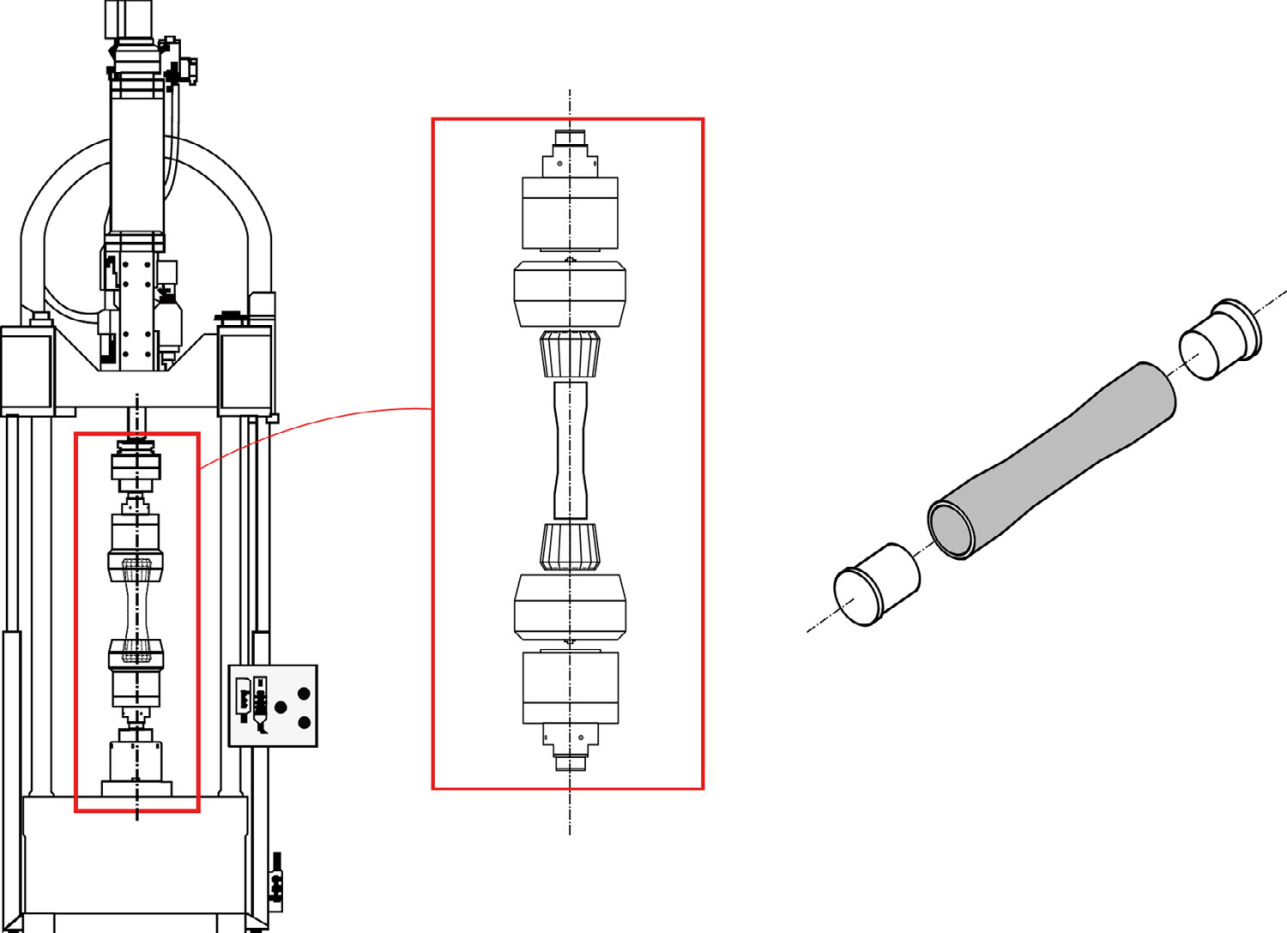
Illustration of the test system and clamping of the CFRP tube test specimens via hydraulically tightened collets for the combined tension/compression-torsion testing, as well as the aluminum inlets for reinforcement in the clamping area of the specimen.

The tube specimens are filament wound with the carbon fibre rovings Tenax®-E HTS45 of the standard module class and with the medium-viscosity, unmodified epoxy resin Araldite LY 556 by Huntsman, which is based on bisphenol-A. The chemical composition of the resin used in the resin bath during the winding process can be found in the chemical composition table in [3]. Also, the cure and post-cure times are listed in the table. The cyclic tension/compression-torsion tests are conducted on the servo-hydraulic Instron®Type 8802 testing machine pictured in Fig. 2. It is equipped with a hydrostatic axial cylinder with 100 kN nominal force, a nominal stroke of ±75 mm and also with a torsion cylinder with 1 kNm nominal torque and a nominal angle of rotation of ±45°. As can be seen on the very right of Fig. 2, the specimens are additionally provided with aluminum inlets with a length of 70 mm at the tube ends, which are intended to prevent buckling under clamping pressure.

**Fig. 3:**
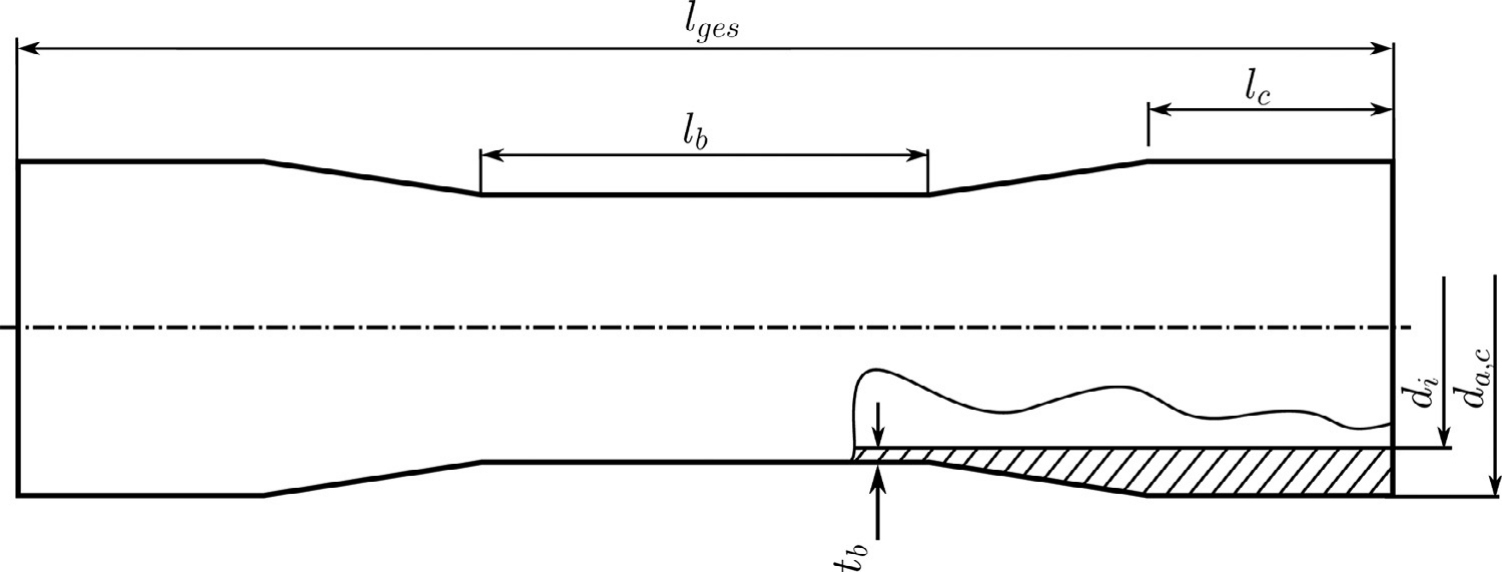
Geometry of the CFRP tube test specimens with information on the geometric dimensions for Table 2.

The specimens are then hydraulically clamped with interchangeable collets for tubular specimens with outer diameters up to 64 mm. The collet system hydraulically clamps the specimen over the entire surface of the tube ends with a length of $l_{c}$ and a clamping pressure of 40 bar for the unidirectional and 60 bar for the multidirectional specimens. Fig. 3 shows the design of the tubular specimen. In line with the typically used force-introducing elements for flat test specimens in the clamping area, the tube test specimens are provided with a thicker wound lay-up in the clamping area. Table 2 shows the average lengths and thicknesses for the three different kinds of lay-ups. The specimens are finally tested up to final failure, where no force is taken by the specimen anymore and the number of endured cycles up to that failure is pointed out in the tables. For a lot more information on the hysteresis loops within the cyclic loading, the data can be found in the mendeley repository [3].

**Table 2:** Average geometrical data for the uni- and multidirectional tube specimen.

Specimen	${\bar{l}}_{ges}$	${\bar{l}}_{b}$	${\bar{l}}_{c}$	${\bar{t}}_{b}$	${\bar{d}}_{i}$	${\bar{d}}_{a,c}$
	mm	mm	mm	mm	mm	mm
Unidirectional	251,7	82	60,4	0,9	60,3	63,6
Angle-Ply	255,2	82	60,5	1,0	60,3	63,3
Multidirectional	260,8	82	65,4	1,1	60,3	63,6

## Ethics Statements

This article provides raw data recorded at the Institute for Automotive Engineering of the University of Applied Sciences in Cologne with the use of specimens produced at the Institute of Polymer Materials and Plastics Engineering of the Clausthal University of Technology. Neither data collected from any social media platform is presented nor does this article involve the use of any human subjects or animal experiments. Therefore, the authors state to have no known conflict with ethics in publishing.

## CRediT authorship contribution statement

**Marc Moeller:** Conceptualization, Methodology, Investigation, Data curation, Formal analysis, Visualization, Writing – original draft. **Jochen Blaurock:** Conceptualization, Supervision, Writing – review & editing. **Gerhard Ziegmann:** Conceptualization, Supervision, Writing – review & editing.

## Declaration of Competing Interest

The authors declare that they have no known competing financial interests or personal relationships that could have appeared to influence the work reported in this paper.

## Data Availability

Raw date a for fatigue dataset for carbon fibre-reinforced polymers under uni- and multiaxial loads with varying biaxiality and stress ratios. Part 1: Proportional multiaxial loads (Original data) (Mendeley Data).

## Appendix Supplementary materials

Image, application 1
