# Supplementary material for: Fatigue dataset for carbon fibre-reinforced polymers under uni- and multiaxial loads with varying biaxiality and proportional stress ratios
Source: Data Brief. 2022 Nov 18;45:108757. doi: 10.1016/j.dib.2022.108757 (PMC9747653; doi:10.1016/j.dib.2022.108757)
Supplement: Supplementary file 1 [file mmc1.pdf]

Table 1: UDXXXP01X90, Uni-axial Torsion, UD (±89,16 ◦), Stress Ratio R = 0.1

No.	SNo.	φ	R_A	R_T	ξ	σ_{max}	τ_{max}	f_A	f_T	N
-	-		-	-	°	MPa	MPa	Hz	Hz	-
U-16	6	0,51	-	0.1	90,0	0,00	47,55	-	3	17226
U-24	9	0,51	-	0.1	90,0	0,00	46,19	-	3	99780
U-28	10	0,53	-	0.1	90,0	0,00	47,55	-	3	26452
U-39	14	0,55	-	0.1	90,0	0,00	43,25	-	3	337421
U-41	15	0,56	-	0.1	90,0	0,00	46,42	-	2	XC-32845
U-43	15	0,55	-	0.1	90,0	0,00	46,42	-	3	65585
U-44	16	0,55	-	0.1	90,0	0,00	54,77	-	3	2316
U-45	16	0,56	-	0.1	90,0	0,00	45,94	-	3	115817
U-48	17	0,55	-	0.1	90,0	0,00	51,57	-	3	10628
U-50	18	0,54	-	0.1	90,0	0,00	47,34	-	3	29573
U-53	19	0,53	-	0.1	90,0	0,00	54,40	-	3	3487
U-55	19	0,58	-	0.1	90,0	0,00	45,94	-	3	XC-39868
U-63	23	0,56	-	0.1	90,0	0,00	47,38	-	3	54706
U-64	23	0,55	-	0.1	90,0	0,00	57,25	-	3	659
U-65	23	0,58	-	0.1	90,0	0,00	47,46	-	3	21222
U-67	24	0,56	-	0.1	90,0	0,00	46,65	-	3	58258
U-80	29	0,51	-	0.1	90,0	0,00	47,46	-	3	48242
U-82	30	0,56	-	0.1	90,0	0,00	42,22	-	3	RO-1598453
U-82b	30	0,56	-	0.1	90,0	0,00	52,28	-	3	HA-6233
U-83	30	0,56	-	0.1	90,0	0,00	50,35	-	3	11446
U-85	31	0,55	-	0.1	90,0	0,00	46,76	-	3	72677
U-86	31	0,57	-	0.1	90,0	0,00	47,24	-	3	68822
U-90	33	0,56	-	0.1	90,0	0,00	46,16	-	3	48230
U-94	35	0,56	-	0.1	90,0	0,00	54,00	-	3	3575
U-96	35	0,58	-	0.1	90,0	0,00	48,33	-	3	17954
U-98	36	0,56	-	0.1	90,0	0,00	50,74	-	3	4643
U-157	58	0,57	-	0.1	90,0	0,00	46,50	-	3	95069

Table 2: UDXXXP05X90, Uni-axial Torsion, UD (±89,16 ◦), Stress Ratio R=0.5

No.	SNo.	φ	R_A	R_T	ξ	σ_{max}	τ_{max}	f_A	f_T	N
-	-		-	-	°	MPa	MPa	Hz	Hz	-
U-107	40	0,54	-	0.5	90,0	0,00	52,19	-	2	2367062
U-210	77	0,49	-	0,5	90,0	0,00	54,27	-	2	RO-1260460
U-212	77	0,51	-	0,5	90,0	0,00	61,61	-	2	24207
U-219	80	0,52	-	0,5	90,0	0,00	53,63	-	2	XC-272024
U-220	80	0,52	-	0,5	90,0	0,00	64,35	-	2	226
U-221	80	0,52	-	0,5	90,0	0,00	61,61	-	2	35999
U-222	81	0,52	-	0,5	90,0	0,00	60,78	-	2	65268
U-223	81	0,50	-	0,5	90,0	0,00	65,23	-	2	935
U-224	81	0,50	-	0,5	90,0	0,00	57,20	-	2	973968

Table 3: UDXXXM1X90, Uni-axial Torsion, UD (±89,16 ◦), Stress Ratio R=-1

No.	SNo.	φ	R_A	R_T	ξ	σ_{max}	τ_{max}	f_A	f_T	N
-	-		-	-	°	MPa	MPa	Hz	Hz	-
U-186	69	0,52	-	-1	90,00	0	33,54	-	1	843
U-188	69	0,55	-	-1	90,00	0	25,76	-	2	21533
U-189	70	0,54	-	-1	90,00	0	18,96	-	2	RO-1655817
U-190	70	0,53	-	-1	90,00	0	33,12	-	1	3714
U-191	70	0,53	-	-1	90,00	0	25,76	-	2	72726
U-192	71	0,54	-	-1	90,00	0	45,50	-	1	201
U-193	71	0,53	-	-1	90,00	0	26,93	-	2	97845
U-194	71	0,53	-	-1	90,00	0	22,08	-	2	83053

Table 4: UDP01XXXX0, Uni-axial Tension, UD (±89,16 ◦), Stress Ratio R=0.1

No.	SNo.	φ	R_A	R_T	ξ	σ_{max}	τ_{max}	f_A	f_T	N
-	-		-	-	°	MPa	MPa	Hz	Hz	-
U-18	7	0,50	0.1	-	0,0	21,17	0,00	8	-	XC-1
U-35	13	0,50	0.1	-	0,0	22,02	0,00	8	-	429888
U-52	18	0,53	0.1	-	0,0	25,15	0,00	8	-	18545
U-58	21	0,55	0.1	-	0,0	23,22	0,00	8	-	51010
U-60	21	0,56	0.1	-	0,0	18,02	0,00	10	-	RO-3345350
U-60b	21	0,56	0.1	-	0,0	22,67	0,00	8	-	HA-25020
U-66	24	0,55	0.1	-	0,0	20,82	0,00	8	-	615931
U-78	29	0,52	0.1	-	0,0	33,53	0,00	8	-	6
U-79	29	0,50	0.1	-	0,0	38,50	0,00	8	-	2
U-91	33	0,56	0.1	-	0,0	23,85	0,00	8	-	XC-8455
U-101	37	0,57	0.1	-	0,0	20,79	0,00	8	-	430842
U-111	41	0,48	0.1	-	0,0	21,99	0,00	8	-	106596
U-114	42	0,48	0.1	-	0,0	21,70	0,00	8	-	138704
U-115	43	0,48	0.1	-	0,0	22,85	0,00	8	-	108512
U-121	45	0,47	0.1	-	0,0	38,29	0,00	8	-	211
U-123	46	0,47	0.1	-	0,0	36,00	0,00	8	-	70
U-125	46	0,47	0.1	-	0,0	31,97	0,00	8	-	3488
U-127	47	0,50	0.1	-	0,0	19,58	0,00	8	-	591628
U-136	51	0,51	0.1	-	0,0	29,69	0,00	8	-	3018
U-138	51	0,51	0.1	-	0,0	26,74	0,00	8	-	9167

Table 5: UDM1XXXX0, Uni-axial Tension, UD (±89,16 ◦), Stress Ratio R=-1

No.	SNo.	φ	R_A	R_T	ξ	σ_{max}	τ_{max}	f_A	f_T	N
-	-		-	-	°	MPa	MPa	Hz	Hz	-
U-195	72	0,52	-1	-	0,00	23,79	0,00	3	-	12780
U-198	73	0,55	-1	-	0,00	24,54	0,00	3	-	2588
U-203	74	0,52	-1	-	0,00	20,84	0,00	3	-	XC-1778
U-204	75	0,51	-1	-	0,00	20,54	0,00	3	-	276310
U-206	75	0,50	-1	-	0,00	20,23	0,00	3	-	31288
U-207	76	0,54	-1	-	0,00	24,88	0,00	3	-	XC-586
U-208	76	0,51	-1	-	0,00	20,82	0,00	3	-	48908
U-209	76	0,52	-1	-	0,00	20,51	0,00	3	-	94317

Table 6: UDP01P01X28, Tension-Torsion, UD (±89, 16◦), Stress Ratio RA =0.1, RT =0.1, ξ = 28◦

No.	SNo.	φ	R_A	R_T	ξ	σ_{max}	τ_{max}	f_A	f_T	N
-	-		-	-	°	MPa	MPa	Hz	Hz	-
U-70	25	0,49	0.1	0.1	27,9	25,27	13,35	5	5	4504
U-72	26	0,49	0.1	0.1	27,8	24,21	12,78	5	5	XC-16
U-97	36	0,58	0.1	0.1	27,9	25,02	13,27	5	5	15686
U-103	38	0,56	0.1	0.1	27,9	27,76	14,70	5	5	3867
U-104	38	0,59	0.1	0.1	27,9	23,96	12,68	5	5	35489
U-116	43	0,50	0.1	0.1	27,9	27,34	14,45	5	5	2717
U-122	45	0,49	0.1	0.1	27,8	22,40	11,81	5	5	158282
U-126	47	0,50	0.1	0.1	27,8	20,47	10,80	5	5	482663
U-133	49	0,49	0.1	0.1	27,9	27,34	14,45	5	5	1178
U-135	50	0,52	0.1	0.1	27,8	21,06	11,12	5	5	XC-69
U-139	52	0,51	0.1	0.1	27,8	24,23	12,80	5	5	47901
U-148	55	0,54	0.1	0.1	27,9	28,15	14,90	5	5	2554
U-150	56	0,52	0.1	0.1	27,8	26,98	14,25	5	5	9032

Table 7: UDP01P01X57, Tension-Torsion, UD (±89, 16◦), Stress Ratio RA =0.1, RT =0.1,ξ = 58◦

No.	SNo.	φ	R_A	R_T	ξ	σ_{max}	τ_{max}	f_A	f_T	N
-	-		-	-	°	MPa	MPa	Hz	Hz	-
U-27	10	0,51	0.1	0.1	57,5	18,81	29,56	5	5	152408
U-32	12	0,56	0.1	0.1	57,7	19,35	30,56	5	5	17481
U-37	13	0,53	0.1	0.1	57,5	15,81	24,85	5	5	1292244
U-95	35	0,56	0.1	0.1	57,9	17,69	28,20	5	5	385654
U-102	38	0,58	0.1	0.1	57,8	18,37	29,23	5	5	XC-2
U-108	40	0,52	0.1	0.1	57,8	17,83	28,30	5	5	250592
U-110	41	0,47	0.1	0.1	57,8	20,17	31,97	5	5	47793
U-112	42	0,48	0.1	0.1	57,8	16,87	26,80	5	5	246892
U-113	42	0,46	0.1	0.1	57,8	20,20	32,07	5	5	58177
U-124	46	0,46	0.1	0.1	57,7	15,52	24,57	5	5	2631246
U-129	48	0,50	0.1	0.1	57,8	16,41	26,04	5	5	174023
U-131	49	0,49	0.1	0.1	57,7	17,08	27,05	5	5	80548
U-134	50	0,51	0.1	0.1	57,7	17,55	27,81	5	5	42883
U-143	53	0,49	0.1	0.1	57,8	19,65	31,18	5	5	32052

Table 8: BAP01P01X19, Tension-Torsion, UD (±70◦), Stress Ratio RA =0.1, RT =0.1,ξ = 19◦

No.	SNo.	φ	R_A	R_T	ξ	σ_{max}	τ_{max}	f_A	f_T	N
-	-		-	-	°	MPa	MPa	Hz	Hz	-
B-3	70	0,55	0.1	0.1	18,9	36,38	12,49	5	5	2424
B-4	70	0,56	0.1	0.1	18,8	26,70	9,09	5	5	27240
B-11	74	0,57	0.1	0.1	19,1	32,65	11,33	5	5	14073
B-12	74	0,54	0.1	0.1	18,9	35,94	12,33	5	5	3974
B-15	76	0,57	0.1	0.1	19,1	31,83	11,03	5	5	46788
B-16	76	0,53	0.1	0.1	18,8	27,73	9,45	5	5	47377
B-17	77	0,54	0.1	0.1	19,1	32,22	11,17	5	5	22154
B-18	77	0,54	0.1	0.1	18,8	27,73	9,45	5	5	80051

Table 9: BAP01P01X49, Tension-Torsion, UD (±70◦), Stress Ratio RA =0.1, RT =0.1,ξ = 49◦

No.	SNo.	φ	R_A	R_T	ξ	σ_{max}	τ_{max}	f_A	f_T	N
-	-		-	-	°	MPa	MPa	Hz	Hz	-
B-2	69	0,57	0.1	0.1	48,7	26,31	29,95	5	5	61672
B-8	72	0,55	0.1	0.1	48,8	29,39	33,60	5	5	6886
B-9	73	0,54	0.1	0.1	48,7	26,31	29,95	5	5	140508
B-10	73	0,53	0.1	0.1	48,8	29,75	34,02	5	5	34721
B-13	75	0,56	0.1	0.1	48,8	30,12	34,44	5	5	27536
B-14	75	0,54	0.1	0.1	48,7	26,29	29,90	5	5	76202

Table 10: MDP01P01X28, Tension-Torsion, UD ([90| ± 70]s), Stress Ratio RA =0.1, RT =0.1,ξ = 28◦

No.	SNo.	φ	R_A	R_T	ξ	σ_{max}	τ_{max}	f_A	f_T	N
-	-		-	-	°	MPa	MPa	Hz	Hz	-
M-6	80	0,52	0.1	0.1	27,7	22,64	11,87	3	3	413909
M-7	81	0,51	0.1	0.1	27,6	22,13	11,59	3	3	432603
M-8	81	0,53	0.1	0.1	28,3	28,85	15,56	3	3	10594
M-9	82	0,52	0.1	0.1	28,3	29,14	15,71	3	3	23165
M-11	83	0,53	0.1	0.1	28,3	28,23	15,21	3	3	9157
M-12	83	0,54	0.1	0.1	28,5	23,19	12,60	3	3	256342
M-14	84	0,53	0.1	0.1	28,5	23,71	12,89	3	3	194601
M-15	85	0,54	0.1	0.1	28,5	23,97	13,04	3	3	140895
M-16	85	0,52	0.1	0.1	28,5	23,69	12,87	3	3	186175
M-18	86	0,53	0.1	0.1	28,5	23,44	12,73	3	3	121945
M-20	87	0,50	0.1	0.1	28,5	23,68	12,85	3	3	105140

Table 11: MDP01P01X67, Tension-Torsion, UD ([90| ± 70]s), Stress Ratio RA =0.1, RT =0.1,ξ = 67◦

No.	SNo.	φ	R_A	R_T	ξ	σ_{max}	τ_{max}	f_A	f_T	N
-	-		-	-	°	MPa	MPa	Hz	Hz	-
M-25	90	0,55	0.1	0.1	67,0	21,41	50,39	3	3	16339
M-28	91	0,54	0.1	0.1	66,9	21,38	50,23	3	3	37914
M-29	92	0,52	0.1	0.1	67,0	18,23	42,91	3	3	235330
M-31	93	0,52	0.1	0.1	67,0	18,03	42,45	3	3	343283
M-37	96	0,53	0.1	0.1	67,0	17,84	41,99	3	3	549806
M-38	96	0,53	0.1	0.1	67,0	21,41	50,39	3	3	67317

Table 12: Chemical Composition

SNo.	LY 556	%-w/wr	Aradur 917	%-w/wr	DY070	%-w/wr	StSe	Cure	Post-Cure
	g	%	g	%	g	%		min.	min.
**6**	216,4	100	193,0	89,19	2,3	1,06	±89,16	90	135
**7**	223,0	100	201,8	90,49	2,4	1,08	±89,16	60	135
**9**	223,5	100	202,2	90,47	2,4	1,07	±89,16	60	135
**10**	254,4	100	228,9	89,98	2,6	1,02	±89,16	60	135
**12**	220,8	100	202,0	91,49	2,3	1,04	±89,16	60	135
**13**	266,5	100	241,7	90,69	2,7	1,01	±89,16	60	135
**14**	231,7	100	212,5	91,71	2,3	0,99	±89,16	60	135
**15**	230,7	100	211,9	91,85	2,5	1,08	±89,16	60	135
**16**	250,8	100	226,6	90,35	2,8	1,12	±89,16	60	135
**17**	232,8	100	210,2	90,29	2,4	1,03	±89,16	60	135
**18**	229,9	100	208,9	90,87	2,3	1,00	±89,16	60	135
**19**	231,8	100	209,2	90,25	2,6	1,12	±89,16	60	135
**21**	257,7	100	235,8	91,50	2,5	0,97	±89,16	60	135
**23**	245,3	100	219,8	89,60	2,0	0,82	±89,16	60	135
**24**	245,1	100	220,6	90,00	2,1	0,86	±89,16	60	135
**25**	192,7	100	174,7	90,66	2,6	1,35	±89,16	60	135
**26**	205,6	100	187,7	91,29	2,2	1,07	±89,16	60	135
**29**	233,9	100	211,6	90,47	2,4	1,03	±89,16	60	135
**30**	273,0	100	242,4	88,79	2,1	0,77	±89,16	60	135
**31**	239,1	100	215,7	90,21	2,3	0,96	±89,16	60	135
**33**	220,7	100	199,3	90,30	2,7	1,22	±89,16	60	135
**35**	245,9	100	221,7	90,16	2,3	0,94	±89,16	60	135
**36**	245,3	100	219,8	89,60	2,4	0,98	±89,16	60	135
**37**	224,4	100	202,2	90,11	2,1	0,94	±89,16	60	135
**38**	239,8	100	216,4	90,24	2,5	1,04	±89,16	60	940
**40**	234,4	100	211,4	90,19	2,6	1,11	±89,16	60	135
**41**	216,9	100	195,2	90,00	2,6	1,20	±89,16	60	135
**42**	251,2	100	226,3	90,09	2,3	0,92	±89,16	60	135
**43**	258,2	100	233,8	90,55	2,6	1,01	±89,16	60	135
**45**	246,4	100	220,9	89,65	2,6	1,06	±89,16	60	135
**46**	224,8	100	202,9	90,26	2,3	1,02	±89,16	60	135
**47**	223,0	100	200,7	90,00	2,3	1,03	±89,16	60	135
**48**	224,0	100	202,1	90,22	2,4	1,07	±89,16	60	135
**49**	269,5	100	251,1	93,17	2,7	1,00	±89,16	60	135
**50**	220,9	100	198,9	90,04	2,4	1,09	±89,16	60	135
**51**	441,8	100	398,3	90,15	4,5	1,02	±89,16	90	135
**52**	441,8	100	398,3	90,15	4,5	1,02	±89,16	60	135
**53**	222,9	100	201,2	90,26	2,3	1,03	±89,16	60	135
**55**	241,9	100	217,7	90,00	2,4	0,99	±89,16	60	135
**56**	223,9	100	199,9	89,28	2,3	1,03	±89,16	60	135
**58**	342,9	100	309,9	90,38	3,5	1,02	±89,16	60	135
**69**	446,3	100	400,6	89,75	4,7	1,05	±70	60	135
**70**	436,3	100	392,9	90,05	4,5	1,03	±70	60	135
**71**	436,3	100	392,9	90,05	4,5	1,03	±70	60	135
**72**	469,9	100	423,3	90,08	4,9	1,05	±70	60	135
**73**	469,9	100	423,3	90,08	4,9	1,05	±70	60	135

**74**	460,7	100	416,6	90,42	4,7	1,03	±70	60	135
**75**	460,7	100	416,6	90,42	4,7	1,03	±70	60	135
**76**	452,8	100	407,9	90,08	4,8	1,06	±70	60	135
**77**	452,8	100	407,9	90,08	4,8	1,06	±70	60	135
**80**	389,0	100	351,4	90,34	4,1	1,05	[90/±70/90]	60	135
**81**	389,0	100	351,4	90,34	4,1	1,05	[90/±70/90]	60	135
**82**	431,1	100	388,8	90,18	4,4	1,03	[90/±70/90]	60	135
**83**	431,1	100	388,8	90,18	4,4	1,03	[90/±70/90]	60	135
**84**	401,6	100	361,4	89,99	4,2	1,04	[90/±70/90]	60	135
**85**	401,6	100	361,4	89,99	4,2	1,04	[90/±70/90]	60	135
**86**	359,5	100	325,5	90,55	3,8	1,05	[90/±70/90]	60	135
**87**	359,5	100	325,5	90,55	3,8	1,05	[90/±70/90]	60	135
**90**	368,1	100	332,2	90,24	4,0	1,09	[90/±70/90]	60	135
**91**	368,1	100	332,2	90,24	4,0	1,09	[90/±70/90]	60	135
**92**	388,2	100	350,9	90,41	4,0	1,04	[90/±70/90]	60	135
**93**	388,2	100	350,9	90,41	4,0	1,04	[90/±70/90]	60	135
**96**	360,7	100	325,4	90,22	3,9	1,08	[90/±70/90]	60	135
